# Genome Analysis and Optimization of Caproic Acid Production of *Clostridium butyricum* GD1-1 Isolated from the Pit Mud of Nongxiangxing Baijiu

**DOI:** 10.4014/jmb.2304.04013

**Published:** 2023-06-26

**Authors:** Min Li, Tao Li, Jia Zheng, Zongwei Qiao, Kaizheng Zhang, Huibo Luo, Wei Zou

**Affiliations:** 1 College of Bioengineering, Sichuan University of Science and Engineering, Yibin, Sichuan 644005, P.R. China; 2 Wuliangye Yibin Co., Ltd., Yibin, Sichuan 644000, P.R. China; 3 Liquor Brewing Biotechnology and Application Key Laboratory of Sichuan Province, Sichuan University of Science and Engineering, Yibin, Sichuan 644005, P.R. China

**Keywords:** Baijiu, pit mud, caproic acid, *Clostridium butyricum*, genome, fermentation optimization

## Abstract

Caproic acid is a precursor substance for the synthesis of ethyl caproate, the main flavor substance of nongxiangxing baijiu liquor. In this study, *Clostridium butyricum* GD1-1, a strain with high caproic acid concentration (3.86 g/l), was isolated from the storage pit mud of nongxiangxing baijiu for sequencing and analysis. The strain’s genome was 3,840,048 bp in length with 4,050 open reading frames. In addition, virulence factor annotation analysis showed *C. butyricum* GD1-1 to be safe at the genetic level. However, the annotation results using the Kyoto Encyclopedia of Genes and Genomes Automatic Annotation Server predicted a deficiency in the strain’s synthesis of alanine, methionine, and biotin. These results were confirmed by essential nutrient factor validation experiments. Furthermore, the optimized medium conditions for caproic acid concentration by strain GD1-1 were (g/l): glucose 30, NaCl 5, yeast extract 10, peptone 10, beef paste 10, sodium acetate 11, L-cysteine 0.6, biotin 0.004, starch 2, and 2.0% ethanol. The optimized fermentation conditions for caproic acid production by *C. butyricum* GD1-1 on a single-factor basis were: 5% inoculum volume, 35°C, pH 7, and 90% loading volume. Under optimal conditions, the caproic acid concentration of strain GD1-1 reached 5.42 g/l, which was 1.40 times higher than the initial concentration. *C. butyricum* GD1-1 could be further used in caproic acid production, NXXB pit mud strengthening and maintenance, and artificial pit mud preparation.

## Introduction

Baijiu is known around the world as a traditional Chinese distilled beverage that has been produced and consumed for millennia [1]. According to its characteristic flavor substances, baijiu can be divided into 12 aroma types. The three main types are nongxiangxing baijiu (NXXB), jiangxiangxing baijiu, and qingxiangxing baijiu [2], with NXXB accounting for more than 70% of China’s total liquor yield [3]. NXXB is made from a mixture of crushed sorghum, wheat, corn, rice, and glutinous rice through a series of processes, including solid-state fermentation, distillation, and storage in mud pits [4, 5]. After successive rounds of brewing production, the pit mud of NXXB forms a special microbial community consisting mainly of bacteria, archaea, and fungi. The microbial communities produce specific metabolites, which give NXXB its unique and intense flavor [6]. More than 1,300 flavor compounds have been identified in NXXB, including ethyl caproate, ethyl lactate, ethyl acetate, and ethyl butyrate [7, 8]. Among them, ethyl caproate is considered to be the main flavor compound in NXXB [2, 9], conferring a pineapple-like aroma [10]. The precursor of ethyl caproate, caproic acid, is a colorless or light yellow, oily liquid that also contributes to the characteristic flavor of NXXB by giving it a pungent, sour and spicy taste [2, 11]. In addition, caproic acid-producing bacteria (CPB) are the main microorganisms that synthesize caproic acid in the Chinese baijiu ecosystem, and they can affect the flavor of baijiu in pit mud [12].

CPB have been widely identified in the NXXB ecosystem. These bacteria include *Clostridium*, *Bacillus*, *Ruminococcaceae*, *Caproiciproducens*, and *Rummeliibacillus* [11, 12]. *Clostridium* that produce caproic acid mainly include *C. kluyveri*, *C. celerecrescens*, *C. tyrobutyricum*, and *C. swellfunianum* [13-15]. The most widely used *Clostridium* caproic acid-producing strain is *C. kluyveri*. This bacterium very efficiently synthesizes caproic acid, but its substrate utilization spectrum is very limited [14]. *C. celerecrescens* produces caproic acid, but only negligibly produces ethyl caproate [16]. Ethyl caproate is often produced by esterification of caproic acid with ethanol produced by *Saccharomyces cerevisiae* [16], or by co-culture with *C. tyrobutyricum* to promote caproic acid production, improving the synthesis of ethyl caproate [17]. Butyric acid is an intermediate for the synthesis of caproic acid by microorganisms [18]. In our previous study, *C. butyricum* strain GD1-1, which produces caproic acid, was isolated from the pit mud of NXXB, and the initial screening concentration of caproic acid was 3.86 g/l. Applying the caproic acid fermentation broth of *C. butyricum* GD1-1 to the pit-filling and rain pits process of NXXB can increase the content of caproic acid in the pit mud. This method has been proved to increase the content of flavor esters [19].

In this study, the caproic acid biosynthesis pathway, carbon source and nitrogen source utilization pathway, and essential nutrient synthesis were investigated by genome sequencing and Kyoto Encyclopedia of Genes and Genomes Automatic Annotation Server (KAAS) annotation [20, 21]. Based on the annotation results, the composition of the caproic acid production medium and fermentation conditions were optimized to further improve the caproic acid production capacity of *C. butyricum* GD1-1, a crucial step for increasing the content of ethyl caproate in NXXB and improving the quality of pit mud.

## Materials and Methods

### Strains and Materials

*C. butyricum* GD1-1 was isolated from the pit mud of NXXB in the early research of our research group [11]. The initial Reinforced Clostridial Medium (RCM, pH 6.8) used as the fermentation medium contained glucose 5 g, NaCl 5 g, sodium acetate 3 g, peptone 10 g, yeast extract 5 g, beef extract 10 g, starch 1 g, L-cysteine hydrochloride 0.5 g, and filter-sterilized ethyl alcohol 20 ml in 1 L distilled water.

Chemically defined medium (CDM) used in this study was the same as in the previous paper [22].

### Bacterial DNA Extraction

A single colony was inoculated into 100 ml RCM, incubated anaerobically at 35°C for 12 h, and cultured for two generations to obtain the seed liquid. This liquid was inoculated (5% v/v) into the centrifuged RCM and incubated at 35°C until the optical density at 600 nm (OD_600nm_) reached 0.6-0.8, indicative of a logarithmic growth phase. The bacteria were collected, centrifuged, and resuspended in phosphate-buffered saline. This process was repeated three times. The genomic DNA of *C. butyricum* GD1-1 was extracted using a Bacterial Genomic DNA Rapid Extraction Kit (Tiangen Biochemical Technology Co., Ltd., China). The extracted genome was sequenced as described below.

### Whole-Genome Sequencing and Annotation

The genome of *C. butyricum* GD1-1 was sequenced by Shanghai Personal Biotechnology Co., Ltd. (Shanghai, China). A 400-bp insert library was prepared using the TruSeq DNA Sample Prep Kit (Illumina, Inc., USA). Sequencing was performed on the HiSeq2000 sequencing system (Illumina) with a 200-cycle paired-end configuration at the National Laboratory of Genomics for Biodiversity (LANGEBIO, Mexico). Libraries with different indices were multiplexed and loaded using an Illumina HiSeq instrument according to the manufacturer’s instructions. The fastQC bioinformatics workflow was used for quality control of high-quality sequencing data [23]. CheckM was used to evaluate the quality of genome assembly of *C. butyricum* GD1-1 [24]. GeneMarkS was used to predict the protein-coding genes of the bacterial genome [25]. tRNAscan-SE was used to predict transfer RNA (tRNA) genes in the whole genome [26]. Barrnap (URL: https://github.com/tseemann/barrnap) was used to predict ribosomal RNA (rRNA) genes. The other noncoding RNAs were mainly predicted by comparison with the rfam database [27]. Genes were annotated by the NCBI Prokaryotic Genome Annotation Pipeline (https://academic.oup.com/nar/article/44/14/6614/2468204?login=false). Metabolic pathways were annotated using KAAS [28]. Clusters of Orthologous Groups of proteins (COG) annotation was performed using the eggNOG-mapper for assigning the COG category to the genes [29]. Virulence factor prediction was performed using VFanalyzer available in the virulence factor database (VFDB) [22].

### Medium Optimization

Medium optimization was performed to identify the most suitable medium for the growth and fermentation of specific production strains. Optimization involved screening and experimentally adjusting the composition and dosage of the medium with the goal of obtaining the maximum yield of fermentation products. The optimal formulation of a medium is necessary to improve the yield of the target product and reduce the production cost [30]. In this study, RCM was used as the basic medium to improve the caproic acid concentration after fermentation of *C. butyricum* GD1-1 by screening and optimizing different carbon and nitrogen sources, nutrients, and inorganic salts, including their concentrations.

### Selection of Carbon Source and Concentration

Based on the results of KAAS annotation, carbon sources in the complete metabolic pathway in *C. butyricum* GD1-1 were selected for validation. These included sodium acetate, glucose, lactose, galactose, fructose, mannose, sucrose, stachyose, melibiose, cellobiose, and starch (Table 1). The caproic acid concentration was detected by individually adding the carbon source (1% w/v) to the fermentation medium and incubating anaerobically at 35°C for 10 days. Based on the resulting growth, the best carbon source was selected. Next, the selected carbon source was added at various concentrations (1.0, 2.0, 3.0, 4.0, and 5.0% w/v) to RCM and the caproic acid concentration was detected after 10 days of anaerobic incubation at 35°C. Based on the resulting caproic acid concentration, the optimal carbon source addition was determined.

### Selection of Nitrogen Source and Concentration

Ammonium chloride, ammonium nitrate, urea, peptone, beef extract, yeast extract, and compound organic nitrogen source (peptone: beef extract: yeast extract = 1:1:1) were added as nitrogen sources in the fermentation medium. The caproic acid content was detected after 10 days of anaerobic incubation at 35°C. After determining the optimal nitrogen source, 0.5, 1.0, 1.5, 2.0, 3.0, or 4.0% of each selected nitrogen source was individually added to the RCM, and the caproic acid content was detected after 10 days of anaerobic incubation at 35°C to determine the optimal nitrogen source addition.

### Screening of Essential Elements and Their Addition Amount

The KAAS annotation results showed that the biosynthetic pathways of three nutrients, alanine, methionine, and biotin, were incomplete in *C. butyricum* GD1-1 (Table 2). *C. butyricum* GD1-1 cannot synthesize these three nutrients by itself, but there is an ATP-binding cassette (ABC) transporter that can transport them into cells for GD1-1 to use. The growth of *C. butyricum* GD1-1 was examined by single deletion or single addition of each of the three nutrients in the CDM to verify whether these three nutrients were synthesis-deficient nutrients for *C. butyricum* GD1-1. An OD_600nm_ < 40% that of the CDM indicated the synthesis-deficient nature of the material [22]. After determining the optimal carbon and nitrogen sources in the RCM, essential nutrients with synthetic defects were added to the RCM based on the single-factor deletion growth experiment to investigate the effect of the essential nutrients in the fermentation medium on the growth and caproic acid production of *C. butyricum* GD1-1. After determining the optimal carbon source, nitrogen source and essential nutrients for the RCM, different factors (NaCl, L-cysteine, biotin, sodium acetate, ethanol and starch) were added to the medium and the caproic acid production of *C. butyricum* GD1-1 was measured after 10 days of anaerobic culture. The amount of each factor was then optimized according to its effect on caproic acid production to investigate the effect of essential nutrients and inorganic salts in the fermentation medium on the caproic acid concentration of *C. butyricum* GD1-1.

### Orthogonal Experiment

The approximate range of caproic acid production by *C. butyricum* GD1-1 was preliminarily determined by single-factor experiments. The significant factors affecting caproic acid production by *C. butyricum* GD1-1 were further optimized by orthogonal experiments. Based on the results of the single-factor experiments, four factors (glucose, peptone, sodium acetate, and ethanol) with significant effects on caproic acid production were selected for the four-level orthogonal experiments (Table 3) to further optimize the levels of factors with significant effects on caproic acid production by strain GD1-1.

### Optimization of Fermentation Conditions

Caproic acid production is a complex microbial process. The concentration of caproic acid can be maximized when the conditions of the reaction system are favorable for the vital activity of caproic acid bacteria. The production of caproic acid is influenced by temperature, pH, and hydrogen partial pressure [31]. The fermentation conditions for caproic acid production by *C. butyricum* GD1-1 were optimized by single-factor (temperature, inoculum, initial pH, loading volume) experiments. The value of each factor that maximized caproic acid production was determined.

### Qualitative and Quantitative Analyses of Caproic Acid

The content of caproic acid in fermentation broth was determined by gas chromatography-mass spectrometry (GC-MS). The fermented broth was centrifuged at 10,000 ×*g* for 20 min. The supernatant was passed through a 0.22-μm organic phase filter membrane and collected in an electropolished pipe. Then, 1 ml of the filtered supernatant was injected into a bottle for testing. The GC conditions were: column, db-wax UI column (30 m) × 0.25 mm, 0.25 μm; temperature set at 40°C with a 1 min holding time followed by an increase to 150°C at a rate 20°C/min, followed by another increase to 250°C at 10°C with a holding time for 2 min; split ratio, 30:1; carrier gas, helium; flow rate, 1 ml/min; flow rate for H2, 40 ml/min; flow rate for O2, 300 ml/min; and use of flame ionization detector. The MS conditions were: electron ionization source, transmission line temperature 250°C, electron energy 70 EV, photomultiplier tube voltage 350 V, and mass scanning range 30-350 amu. For qualitative and quantitative analyses, the MS data obtained by the GC-MS analysis were used to search the 17 standard libraries of the National Institute of Standards and Technology. The content of caproic acid in the fermentation broth was determined using the external standard method.

### Data Processing

Origin software was used to process the experimental data and draw graphs. IBM SPSS Statistics (version R24.0.0.0) was used to conduct variance analysis between the experimental groups. A *p*-value < 0.05 was considered statistically significant.

## Results and Discussion

### Genome Properties of *C. butyricum* GD1-1

**Sequencing Data Collation and Quality Control**

The statistical results of the original sequencing data of *C. butyricum* GD1-1 are shown in the Additional File. The quality (Q) value is the integer mapping result of the base reading error rate p. According to the collation and quality evaluation of the sequencing data of GD1-1, the Q value was calculated to be 40, which was converted to a sequencing error rate of 0.01%. Therefore, the accuracy of the sequencing results of GD1-1 can be guaranteed.

**Fig. 1:**
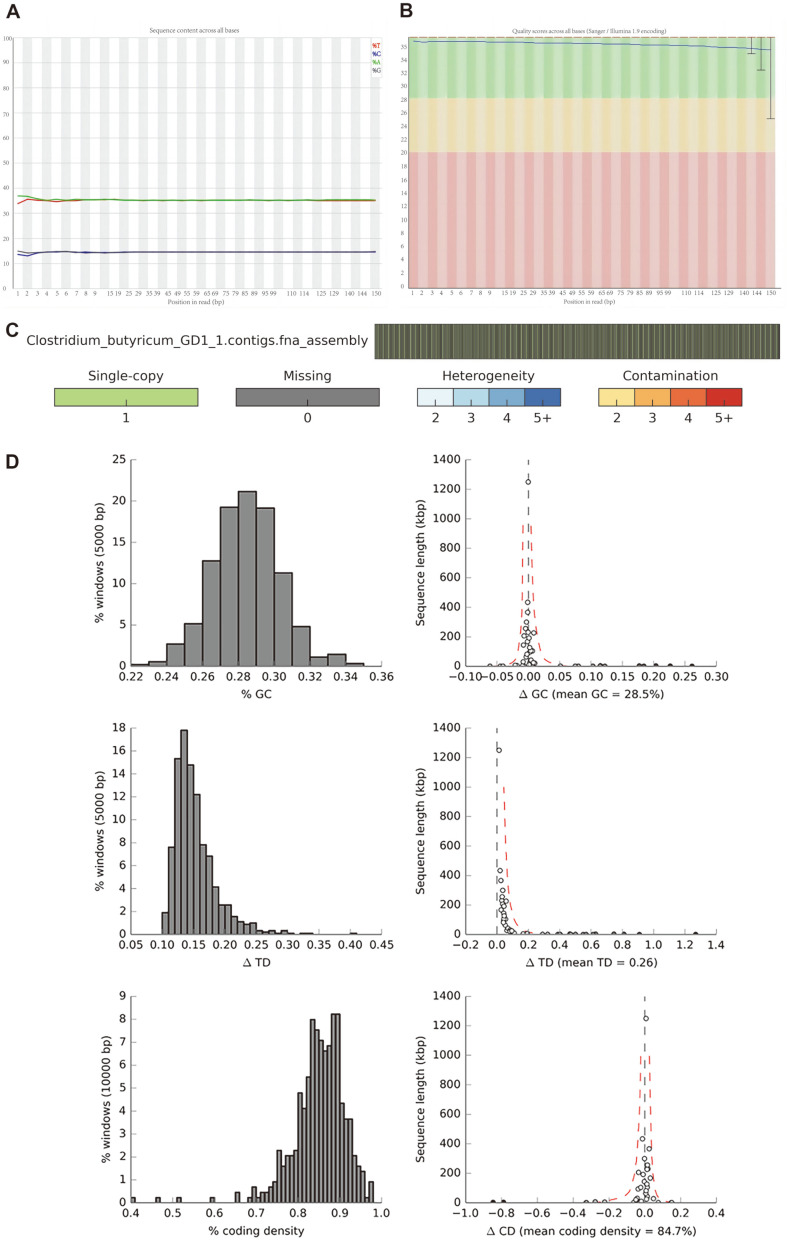
Quality control of sequencing data (A, B) and quality evaluation of genome assembly (C, D) of *Clostridium butyricum* GD1-1.

The quality control results of sequencing data are shown in Figs. 1A and 1B. In the fragmented genome, several AT and GC in the front position were separated. In the other positions, the frequency of the four bases was relatively consistent, indicating the good uniformity of GD1-1 construction and sequencing, as well as the suitability for subsequent information analysis.

The quality assessment results of *C. butyricum* GD1-1 genome assembly are shown in Figs. 1C and 1D. After assembly, the completeness of the *C. butyricum* GD1-1 genome reached 99.19%, and the contamination was 0%. The findings indicated the good integrity and accuracy of the assembly results of the *C. butyricum* GD1-1 genome sequence.

### Genome Assembly and Annotation of *C. butyricum* GD1-1

To obtain a comprehensive understanding of the genetic background of *C. butyricum* GD1-1, we sequenced and annotated its genome (Table 4). The *C. butyricum* genome is 3,840,048 bp in length, has a GC content of 29.64%and contains 4,050 open reading frames, three rRNA genes, and 49 tRNA genes. The nucleotide sequence of *C. butyricum* GD1-1 has been deposited in the NCBI database with the accession number PRJNA759372.

**Fig. 2:**
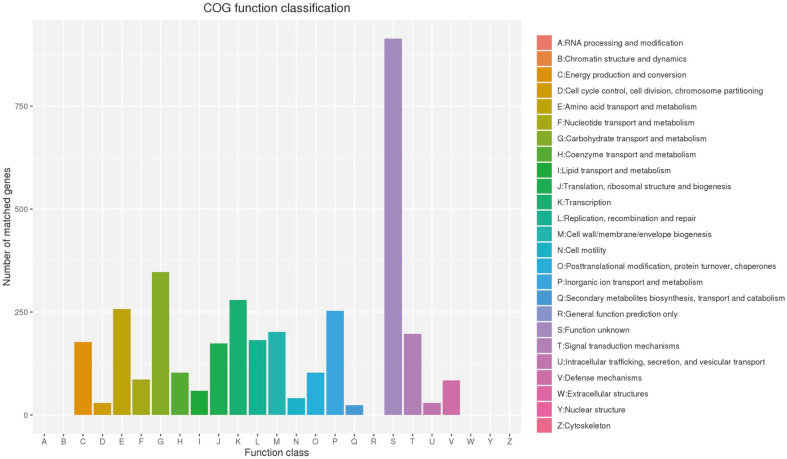
COG function classification of *C. butyricum* GD1-1.

**Fig. 3:**
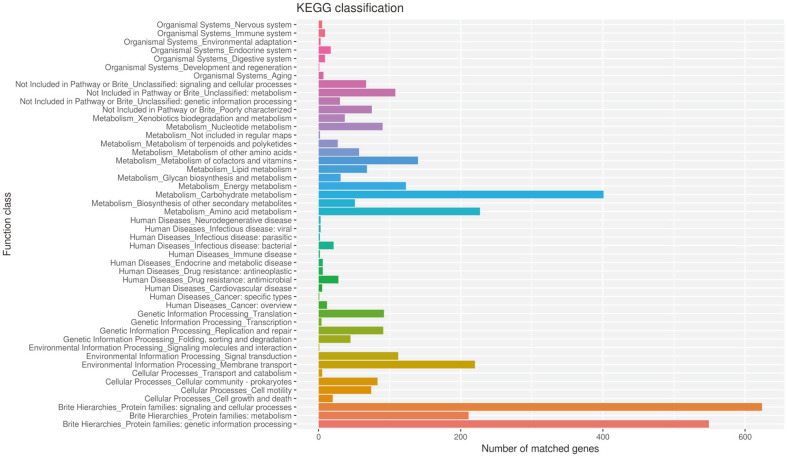
KEGG functional classification of *C. butyricum* GD1-1.

For the COG annotation (Fig. 2), a total of 3,540 genes were assigned with COG terms. These genes accounted for 87.4% of the total genes in *C. butyricum* GD1-1. Function unknown (S) was the largest category (22.56%). Apart from S, carbohydrate transport and metabolism (G) and transcription (K) were the largest two groups, accounting for 8.5% and 6.8%, respectively. A total of 3,805 genes were assigned with KEGG Orthology terms according to the KAAS annotation. The largest three metabolic pathway categories in the genome of strain GD1-1 were carbohydrate metabolism (401), amino acid metabolism (221), and energy metabolism (140) (Fig. 3).

### Caproic Acid Biosynthesis Pathway Analysis

**Fig. 4:**
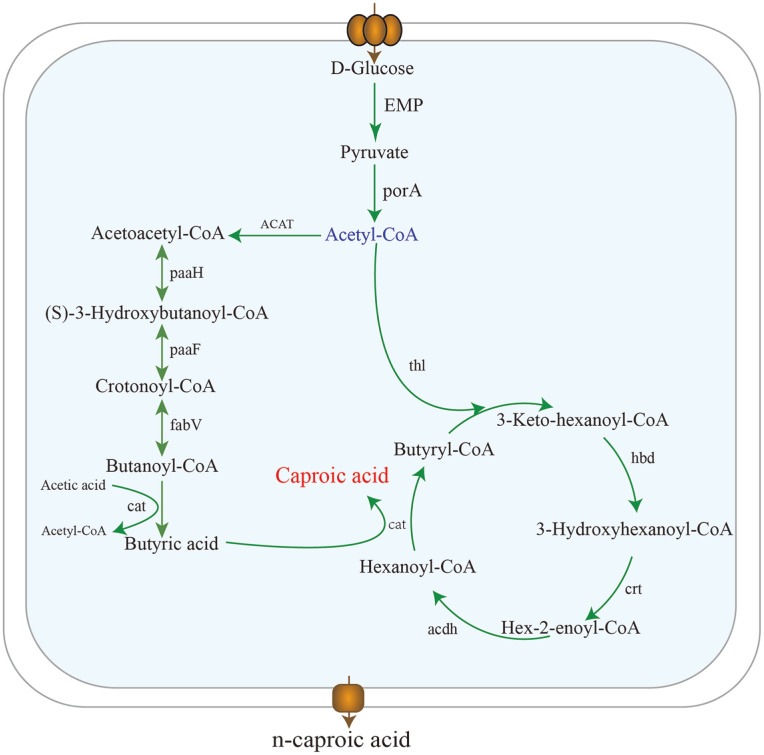
The biosynthetic pathway of caproic acid and other organic acids in *C. butyricum* GD1-1.

Based on the results of KAAS annotation, a three-step process for synthesis of caproic acid by *C. butyricum* GD1-1 using glucose was constructed. In the first step, *C. butyricum* GD1-1 produced acetyl-coenzyme A (CoA) through pyruvic acid by pyruvate ferredoxin oxidoreductase (porA). In the second step, acetyl-CoA was converted to butanoyl-CoA. Butanoyl-CoA and acetic acid were converted to butyric acid and acetyl-CoA by the action of acetyl-CoA transferase. In the third step, hexanoyl-CoA and butyric acid were converted to caproic acid and butyryl-CoA by acyl-CoA transferase. Because of the broad catalytic activity of acyl-CoA transferase, hexanoyl-CoA may also react directly with acetic acid to synthesize caproic acid [32, 33]. The main enzymes involved in the above three steps were successfully annotated (Fig. 4).

### Biosynthesis and Transport of Amino Acids and Vitamins

As shown by the KAAS annotated metabolic pathway map, *C. butyricum* GD1-1 could produce various amino acids, but not alanine and methionine. Moreover, *C. butyricum* GD1-1 could synthesize thiamine, riboflavin, niacin, pantothenic acid, folic acid, and other B vitamins, but not biotin (Table 2).

### Prediction of Virulence Factors

The whole gene protein sequence of *C. butyricum* GD1-1 was analyzed for strain safety by the VFanalyzer analysis program against the Virulence Factors of Pathogenic Bacteria Database (VFDB). The analysis showed that the *C. butyricum* GD1-1 genome did not contain genes related to toxins, anti-phagocytosis, and exoenzyme virulence factors, but did contain genes encoding adhesion-related virulence factors (*FbpA*, *groEL*, *wbpI*, *pebA*). However, some studies have implicated *C. butyricum* as having roles in disease resistance, immunity, inhibition of pathogens, and their adhesion to cells [34, 35]. There was no direct evidence that these genes are pathogenic [36, 37], and therefore *C. butyricum* GD1-1 was considered a safe strain at the genetic level (Table 5).

### Verification of Essential Nutrition Deficiency in *C. butyricum* GD1-1

**Fig. 5:**
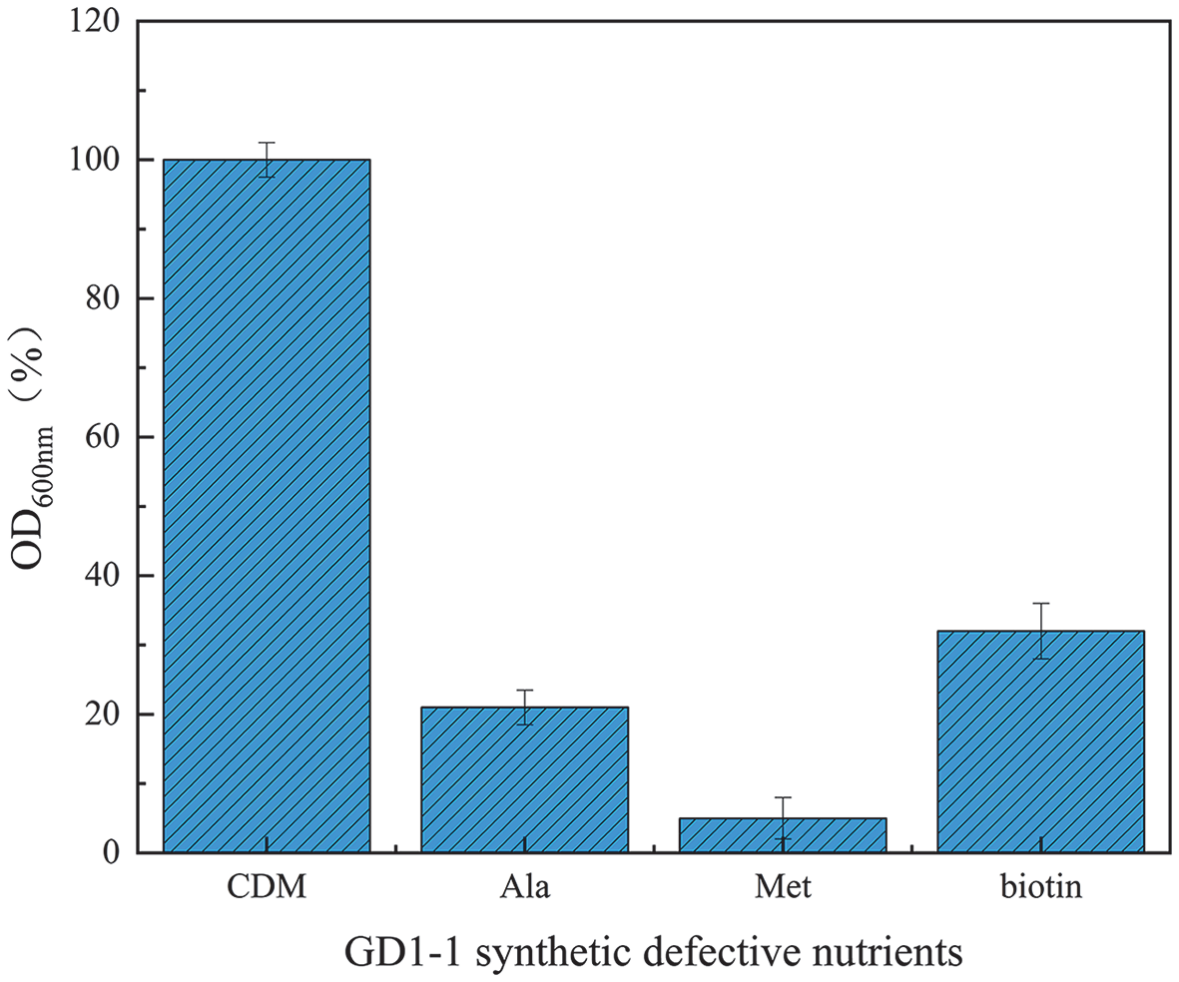
Single-factor growth experiments for *C. butyricum* GD1-1.

To verify whether alanine, methionine, and biotin are synthetic defective nutrients of *C. butyricum* GD1-1, we performed single-factor deletion experiments using the CDM that supported growth. After individually omitting L-alanine, L-methionine, and biotin from CDM, the *C. butyricum* GD1-1 biomass decreased to 21, 5, and 32% that of the control (Fig. 5), respectively, which was lower than 40% of the CDM. The single-factor deletion experiment results were consistent with the KAAS annotation results, confirming that *C. butyricum* GD1-1 was defective in the synthesis of the three nutrients.

### Medium Optimization for Caproic Acid Production Based on KAAS Annotation

**Effects of Different Carbon Sources on Caproic Acid Production**

**Fig. 6:**
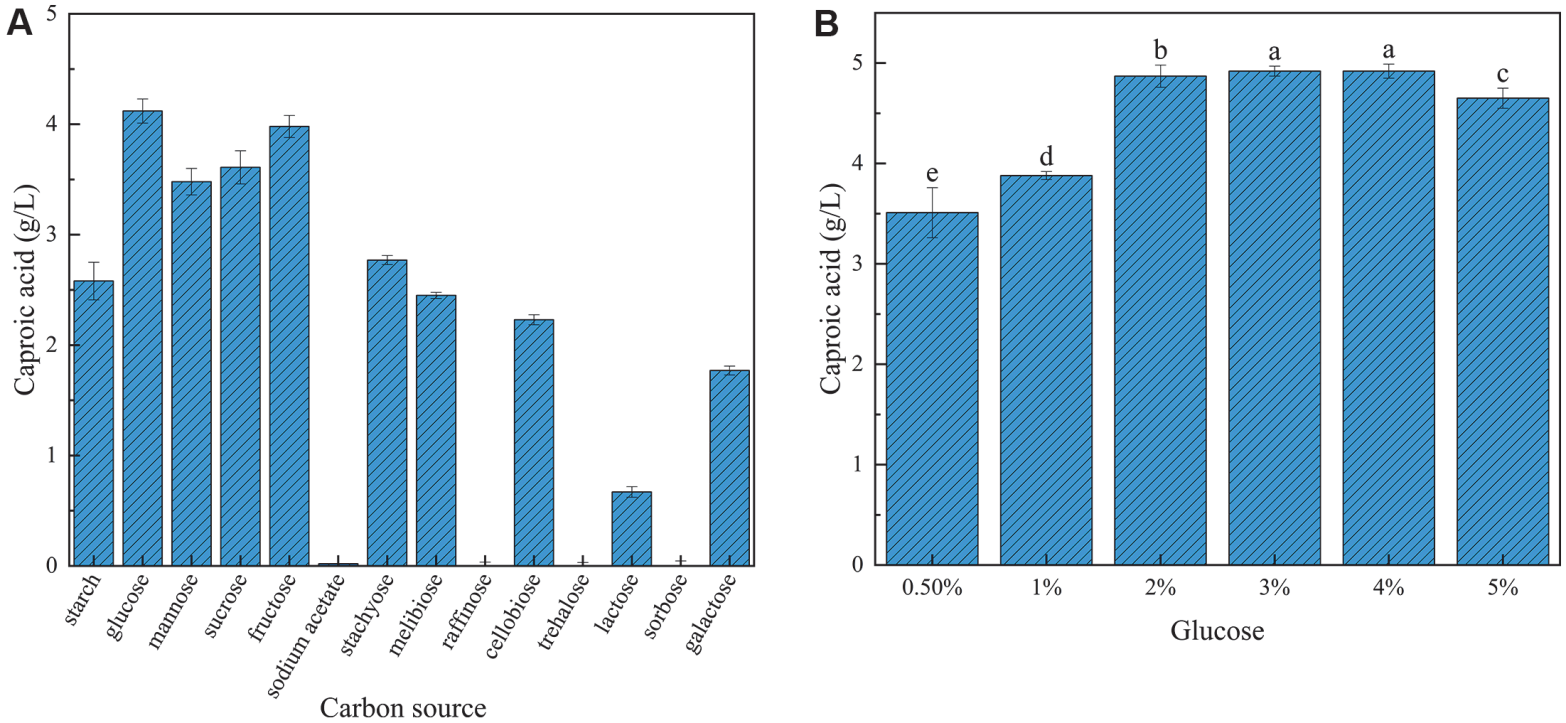
Caproic acid concentration of strain GD1-1 under different carbon sources.

The best carbon source for caproic acid production by strain GD1-1 was glucose (4.12 ± 0.18 g/l), followed by fructose and sucrose (Fig. 6A). When sodium acetate was the sole carbon source, almost no caproic acid was produced. GD1-1 was unable to produce caproic acid using raffinose, trehalose, and sorbose. *C. butyricum* GD1-1 can however use a variety of carbon sources as substrates to produce caproic acid, and has a broad substrate utilization spectrum for caproic acid production. As shown in Fig. 6B, the caproic acid concentration of *C. butyricum* GD1-1 increased and then decreased with the increase of glucose addition. The highest caproic acid concentration was achieved when the addition level reached 3.0%, and therefore the optimal addition level of glucose was 3.0%.

### Effects of Different Nitrogen Sources on Caproic Acid Production

**Fig. 7:**
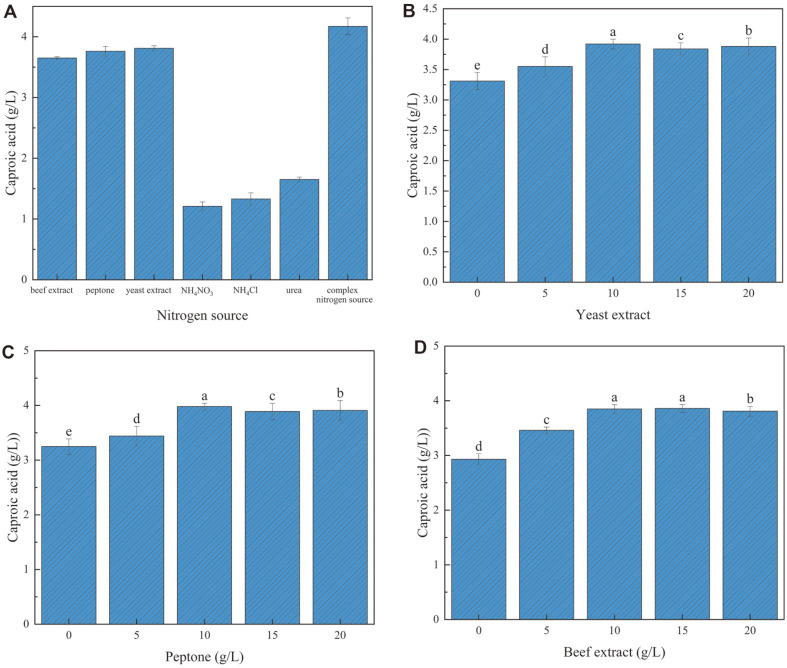
Caproic acid concentration of strain GD1-1 under different nitrogen sources.

The caproic acid production ability of *C. butyricum* GD1-1 using an organic nitrogen source was better than that of an inorganic nitrogen source. The compound organic nitrogen source composed of yeast extract, peptone, and beef extract (1:1:1) effectively promoted the caproic acid concentration of *C. butyricum* GD1-1 and was selected as the optimal nitrogen source (Fig. 7A). The complex organic nitrogen source consisting of yeast extract, peptone, and beef extract was rich in protein, amino acids, vitamins, and trace elements, which could be used to promote the fermentation acid production of *C. butyricum* GD1-1. As shown in Figs. 7B-7D, the value points of yeast extract, peptone, and beef extract were 10 g/l.

### Effects of Essential Nutrients on Growth and Caproic Acid Concentration of *C. butyricum* GD1-1

The results of the single-factor growth experiment confirmed some defects of *C. butyricum* GD1-1 in the synthesis of biotin, L-alanine, and L-methionine. Therefore, these three synthesis-deficient nutrients were used as candidate additions to *C. butyricum* GD1-1 for optimal synthesis of caproic acid. After determining the optimal carbon and nitrogen sources for *C. butyricum* GD1-1, biotin, L-alanine, and L-methionine were added to RCM to explore their effects on the growth and caproic acid production of *C. butyricum* GD1-1.

**Fig. 8:**
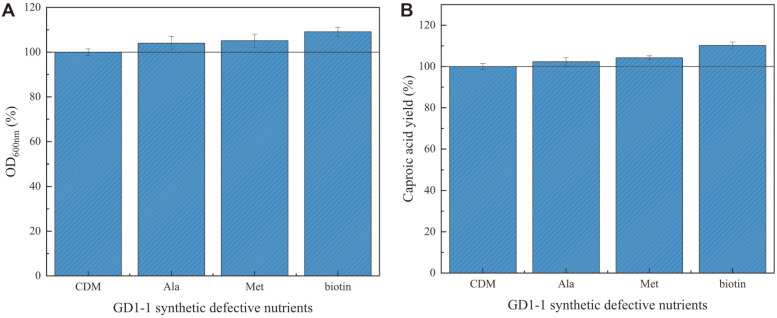
Effects of the addition of essential nutrients on cell growth and caproic acid concentration. (**A**) GD1-1 OD_600nm_; (**B**) GD1-1 caproic acid percentage content.

The OD_600nm_ and caproic acid concentration of *C. butyricum* GD1-1 increased most significantly in RCM supplemented with biotin, with respective values of 9.1% and 10.2% after 10 days of fermentation compared with the control group. In the medium with L-alanine and L-methionine, the OD_600nm_ increased less (4% and 5.1%, respectively) and caproic acid concentration increased by 2.3% and 4.2%, respectively, compared to the control. *C. butyricum* GD1-1 displayed the greatest ability to synthesize caproic acid using biotin metabolism in terms of growth and caproic acid concentration. Biotin has been shown to play an essential role in the production of caproic acid by CPB [38]. In the absence of biotin, the strain may not produce caproic acid. Considering all aspects, only biotin was added in the subsequent optimization experiments of the fermentation medium (Fig. 8).

### Effect of Fermentation Medium of *C. butyricum* GD1-1 on Caproic Acid Concentration

**Fig. 9:**
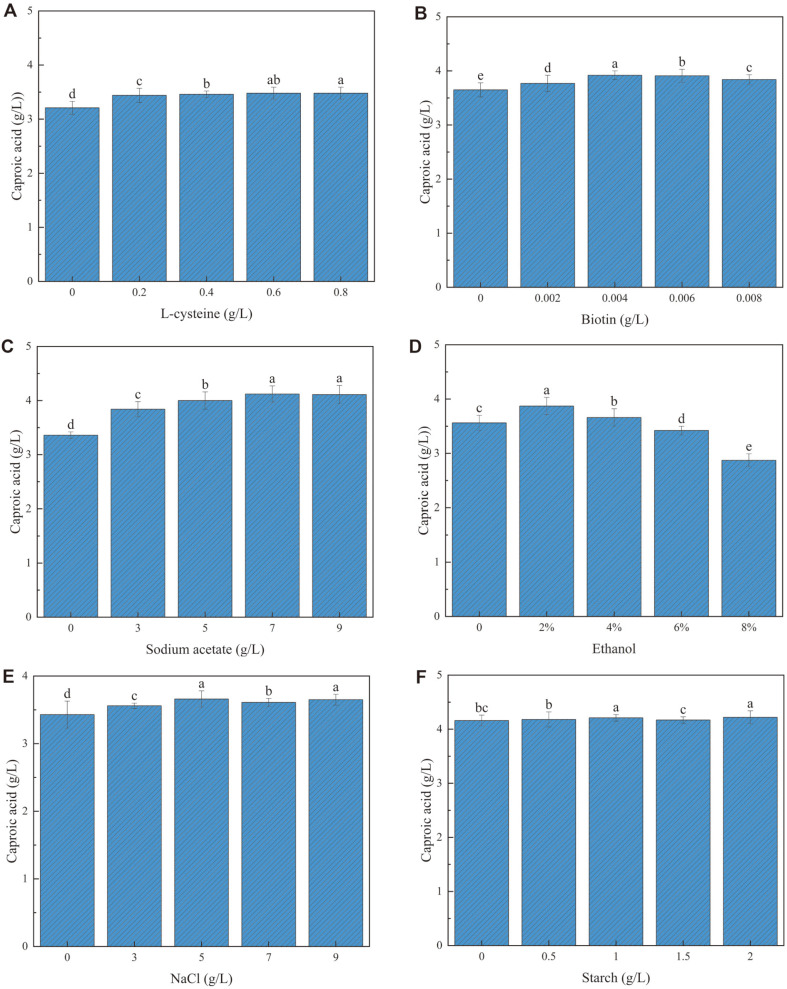
Effects of different factors of L-cysteine (**A**), biotin (**B**), sodium acetate (**C**), ethyl alcohol (**D**), NaCl (**E**), starch (**F**) in RCM on the concentration of caproic acid in GD1-1.

In addition to being an essential amino acid for microbial growth, L-cysteine can also act as a reducing agent. Thus, adding a small amount of L-cysteine in the anaerobic experiment reduced the redox potential of the system [39]. The optimal level of L-cysteine was 0.6 g/l (Fig. 9A). A type of B vitamin, biotin is usually bound to enzymes in microbial metabolism and is also involved in the fixation and carboxylation of carbon dioxide cells [40]. In the present study, the dose of biotin had a significant effect on processes such as metabolism, growth, and reproduction of microbial cells [41] (Fig. 9B). The optimum addition of biotin was 0.004 g/l. The addition of 7 g/l sodium acetate in RCM more effectively improved the caproic acid concentration of *C. butyricum* GD1-1. The promotion effect was not obvious when the sodium acetate level exceeded 7 g/l (Fig. 9C). Therefore, the optimal addition amount of sodium acetate was determined to be 7 g/l. Ethanol could be used as an electron donor for caproic acid bacteria, and the use of a low concentration of ethanol as an electron donor was conducive to a continuous yield of caproic acid [32, 42, 43] (Fig. 9 D). The highest concentration of caproic acid was produced by this strain when ethanol was added at 2.0%. Ethanol and acetate are the most favorable substrates for the synthesis of caproate esters by caproic acid in *Clostridium perfringens*, which can create a synergistic effect to maximize the chain elongation process of caproic acid production [42, 44, 45]. NaCl was indispensable in RCM. Although sodium ions are not involved in cell composition, they are related to the maintenance of cellular osmotic pressure (Fig. 9E). The optimal level of NaCl added was 5 g/l. The results in Fig. 8 show that the following levels enable *C. butyricum* GD1-1 to maintain the optimal growth state and obtain the maximum caproic acid concentration. The findings presented in Fig. 9 indicated the following levels for maximum caproic acid concentration: L-cysteine 0.6 g/l, biotin 0.004 g/l, sodium acetate 7 g/l, ethanol 2.0%, NaCl 5 g/l, and starch 2 g/l.

### Orthogonal Experiment

Based on the results of the single-factor optimization experiment, an L_16_(4^4^) orthogonal experiment was designed to further investigate the interaction between glucose (factor A), peptone (factor B), sodium acetate (factor C), and ethanol (factor D), with four levels of each factor analyzed. The results of the orthogonal experiment are shown in Tables 6 and 7. Based on the experimental results shown in Table 6, the Kn and R values were calculated. The optimal fermentation scheme was A_3_B_2_C_4_D_2_, representing glucose 30 g/l, peptone 10 g/l, sodium acetate 11 g/l, and ethanol 2.0%. Glucose was the most significant factor. The results of the orthogonal experiment analysis of variance are shown in Table 7. Glucose, ethanol, peptone, and sodium acetate all had significant effects on the production of caproic acid. The effect of glucose was most significant (*p* < 0.005). Glucose and peptone provided the necessary carbon and nitrogen sources for the growth of *C. butyricum* GD1-1 to ensure the good growth of the strain. The synergistic effect of ethanol and sodium acetate maximizes the chain elongation reaction of caproic acid production by strain GD1-1 [44]. The optimal medium compositions for caproic acid production by GD1-1 determined in single-factor and orthogonal experiments were: glucose 30 g/l, NaCl 5 g/l, yeast extract 10 g/l, peptone 10 g/l, beef extract 10 g/l, sodium acetate 11 g/l, L-cysteine 0.6 g/l, biotin 0.004 g/l, ethanol 2.0%, and soluble starch 2 g/l. The concentration of caproic acid was 5.29 g/l after 9 days of fermentation at an initial pH of 6.8 and 35°C, which was 1.37 times higher than that before optimization.

### Optimization of Fermentation Conditions

**Fig. 10:**
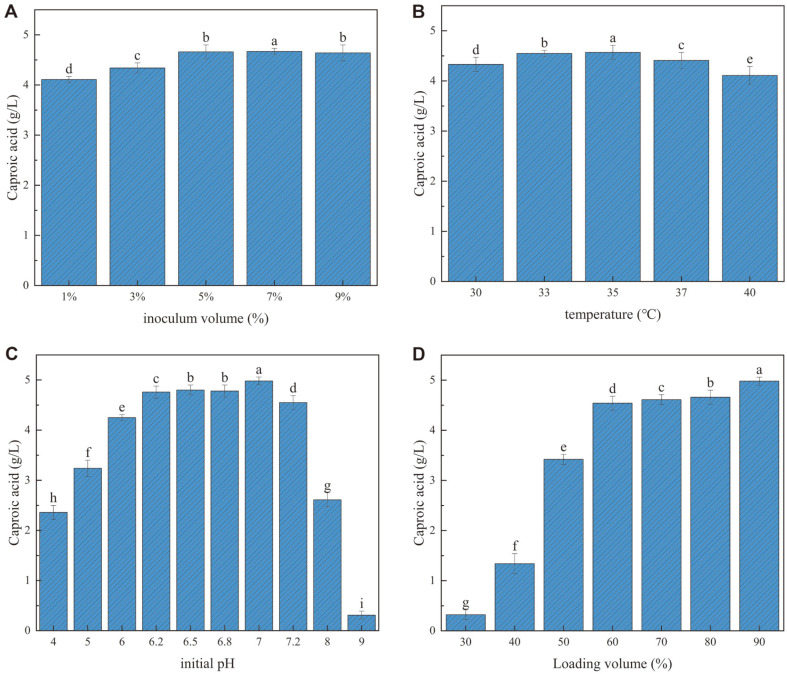
Effects of different fermentation conditions of inoculum volume (**A**), temperature (**B**), initial pH (**C**), and loading volume (**D**) on the concentration of caproic acid in GD1-1.

Based on the optimized *C. butyricum* GD1-1 fermentation medium, the fermentation conditions for caproic acid production were optimized by single-factor tests (inoculum volume, temperature, initial pH, loading volume). In the same medium, the caproic acid concentration of *C. butyricum* GD1-1 increased with increasing inoculum volume. The highest caproic acid concentration was achieved using a 7% inoculum volume (Fig. 10A). In the test temperature gradient, the caproic acid concentration of *C. butyricum* GD1-1 increased and then decreased with increasing temperature, with the highest caproic acid concentration achieved at 35°C (Fig. 10B). In the set pH range, the caproic acid concentration of strain *C. butyricum* GD1-1 increased and then decreased with increasing pH. The production of caproic acid was better in neutral conditions (Fig. 10C). Caproic acid concentration by GD1-1 increased with increasing loading volume, with a peak at 90% loading volume (Fig. 10D). The collective findings supported the following optimal fermentation conditions for *C. butyricum* GD1-1: 7%inoculum, 35°C, pH 7, and 90% loading volume (Fig. 10).

The optimal medium compositions for caproic acid production by *C. butyricum* GD1-1 were glucose 30 g/l, NaCl 5 g/l, yeast extract 10 g/l, peptone 10 g/l, beef extract 10 g/l, sodium acetate 11 g/l, L-cysteine 0.6 g/l, biotin 0.004 g/l, ethanol 2.0%, and soluble starch 2 g/l. In the optimal medium, the pH value was adjusted to 7, loading volume to 90%, and the anaerobic fermentation was performed at 35°C for 9 days. The concentration of caproic acid was 5.42 g/l, which was 1.40 times higher than the initial concentration.

The CPB in the pit mud mainly included *C. kluyveri*, *Caproiciproducens galactitolivorans*, *Rummeliibacillus suwonensis*, *Bacillus*, *Eubacterium pyruvativorans*, and *Ruminococcaceae*. The obligate anaerobic *C. kluyveri* is a functional microorganism that can synthesize important flavor substances, such as caproic acid and ethyl caproate. However, *C. kluyveri* can only synthesize fatty acids with alcohols and acids as substrates, and the substrate utilization spectrum is limited [14, 46]. *C. butyricum* GD1-1 has a wide range of caproic acid production substrates. Based on genome analysis and optimization of the culture medium, the caproic acid yield of R. suwonensis 3B-1 was reported to increase from 4.064 to 4.627 g/l using glucose as substrate by Liu *et al*. In the present work, the same optimization improved the acid production by *C. butyricum* GD1-1, with the caproic acid concentration increased from 3.86 to 5.29 g/l [11]. Sun *et al*. co-cultured *C. fermenticellae* JN500901 and *Novisyntrophococcus fermenticellae* JN500902 to produce caproic acid, and the concentration of caproic acid reached 1.03 g/l after 48 h fermentation. The authors described a significantly higher concentration of caproic acid than that of pure culture [18]. These previous findings could provide a new development direction for *C. butyricum* GD1-1 involving the use of a co-culture system to increase caproic acid concentration.

## Conclusion

In this study, the high caproic acid-producing strain, *C. butyricum* GD1-1, screened from pit mud of NXXB, was used for genome annotation analysis and optimization of fermentation medium conditions for caproic acid production. Further optimization of the fermentation conditions resulted in a caproic acid concentration by strain GD1-1 of 5.42 g/l, which was better than that by most CPB. In addition, *C. butyricum* GD1-1 was able to produce caproic acid using a variety of carbon and inorganic nitrogen sources, indicating the good potential for the bacterium in caproic acid production. *C. butyricum* GD1-1 could be further used to improve the quality of NXXB, pit mud maintenance, and to accelerate the preparation of liquid or artificial pit mud.

## Figures and Tables

**Table 1:** Utilization of carbon and nitrogen sources by *C. butyricum* GD1-1.

	Tested source	Utilization
Carbon source	Glucose	+
	Fructose	+
	Mannose	+
	Galactose	+
	Starch	+
	Sucrose	+
	Stachyose	+
	Melibiose	+
	Raffinose	-
	Cellobiose	+
	Trehalose	-
	Lactose	+
	Sorbose	-
Nitrogen source	Nitrogen	+
	Nitric acid	+
	Nitrous acid	+

Note: ‘+’ represents capable, ‘-’ represents incapable

**Table 2:** Completeness of the biosynthesis pathway of amino acid and vitamins in *C. butyricum* based on KAAS annotation.

Nutritional ingredient	*C. butyricum* GD1-1
Valine	+
Leucine	+
Isoleucine	+
Lysine	+
Arginine	+
Phenylalanine	+
Tyrosine	+
Tryptophan	+
Glycine	+
Alanine	-
Methionine	-
Proline	+
Serine	+
Tyrosine	+
Cysteine	+
Aspartic acid	+
Threonine	+
Glutamic acid	+
Histidine	+
Asparagine	+
Glutamine	+
Thiamine	+
Riboflavin	+
Nicotinic acid	+
Pantothenic acid	+
Folic acid	+
Biotin	-

Note: ‘+’ represents complete, ‘-’ represents incomplete

**Table 3:** Orthogonal experiment involving four factors and four levels of each factor.

Factor	Factor A(glucose%)	Factor B(peptone g/l)	Factor C(sodium acetate g/l)	Factor D(ethyl alcohol %)
1	1	5	5	1
2	2	10	7	2
3	3	15	9	3
4	4	20	11	4

**Table 4:** Genome characteristics of *C. butyricum* GD1-1.

Features	GD1-1
Genome size	3840048 bp
G+C content	29.64%
ORF number	4050
ORF density	0.890 genes per kb
ORF average length	948.16 bp
Intergenic region length	710,045 bp
Coding percentage	84.39%
rRNA	3
tRNA	49
ncRNA	206

**Table 5:** Virulence factor-related coding genes.

Virulence factor class	Related genes
Adherence	*FbpA*; *groEL*; *wbpI*; *pebA*
Antiphagocytosis	——
Biofilm formation	——
Toxins	——
Exoenzyme	——

**Table 6:** Results of L_16_ (4^4^) orthogonal experiment of GD1-1.

	Glucose	Peptone	Sodium acetate	Ethyl alcohol	Caproic acid(g/l)
	1	1	1	1	3.81
	1	2	2	2	4.04
	1	3	3	3	3.72
	1	4	4	4	3.55
	2	1	2	3	4.32
	2	2	1	4	4.21
	2	3	4	1	4.67
	2	4	3	2	4.51
	3	1	3	4	3.89
	3	2	4	3	4.57
	3	3	1	2	4.62
	3	4	2	1	4.81
	4	1	4	2	4.21
	4	2	3	1	4.41
	4	3	2	4	3.71
	4	4	1	3	4.02
K1	3.78	4.058	4.165	4.425	
K2	4.428	4.308	4.22	4.345	
K3	4.473	4.18	4.133	4.158	
K4	4.088	4.222	4.25	3.84	

**Table 7:** Variance analysis of orthogonal experiment of strain GD1-1.

Variance source	Sum of squares	Freedom	Mean square	F value	P value	Significance
Correction model	2.234a	12	0.186	22.191	0.013	
intercept	281.149	1	281.149	33511.683	0.000	
Factor A	1.259	3	0.420	50.031	0.005	*
Factor B	0.130	3	0.043	5.166	0.105	
Factor C	0.345	3	0.011	1.338	0.408	
Factor D	0.811	3	0.270	32.229	0.009	*
error	0.025	3	0.008			
total	283.408	16				
Corrected total	2.259	15				
